# One-Stage Detection without Segmentation for Multi-Type Coronary Lesions in Angiography Images Using Deep Learning

**DOI:** 10.3390/diagnostics13183011

**Published:** 2023-09-21

**Authors:** Hui Wu, Jing Zhao, Jiehui Li, Yan Zeng, Weiwei Wu, Zhuhuang Zhou, Shuicai Wu, Liang Xu, Min Song, Qibin Yu, Ziwei Song, Lin Chen

**Affiliations:** 1 Department of Biomedical Engineering, Faculty of Environment and Life, Beijing University of Technology, Beijing 100124, China; 2 Department of Geriatrics, The Third Medical Center of Chinese PLA General Hospital, Beijing 100039, China; 3 State Key Laboratory of Cardiovascular Disease, Department of Cardiac Surgery, National Center for Cardiovascular Diseases, Fuwai Hospital, Chinese Academy of Medical Sciences, Peking Union Medical College, Beijing 100037, China; 4 Department of Research Center, Shanghai United Imaging Intelligence Co., Ltd., Shanghai 201807, China; 5 College of Biomedical Engineering, Capital Medical University, Beijing 100069, China; 6 State Key Laboratory of Cardiovascular Disease, Department of Structural Heart Disease, National Center for Cardiovascular Diseases, Fuwai Hospital, Chinese Academy of Medical Sciences, Peking Union Medical College, Beijing 100037, China

**Keywords:** coronary angiography, deep learning, coronary artery stenosis detection, convolutional neural network, one-stage detection, without segmentation

## Abstract

It is rare to use the one-stage model without segmentation for the automatic detection of coronary lesions. This study sequentially enrolled 200 patients with significant stenoses and occlusions of the right coronary and categorized their angiography images into two angle views: The CRA (cranial) view of 98 patients with 2453 images and the LAO (left anterior oblique) view of 176 patients with 3338 images. Randomization was performed at the patient level to the training set and test set using a 7:3 ratio. YOLOv5 was adopted as the key model for direct detection. Four types of lesions were studied: Local Stenosis (LS), Diffuse Stenosis (DS), Bifurcation Stenosis (BS), and Chronic Total Occlusion (CTO). At the image level, the precision, recall, mAP@0.1, and mAP@0.5 predicted by the model were 0.64, 0.68, 0.66, and 0.49 in the CRA view and 0.68, 0.73, 0.70, and 0.56 in the LAO view, respectively. At the patient level, the precision, recall, and *F*_1_ *scores* predicted by the model were 0.52, 0.91, and 0.65 in the CRA view and 0.50, 0.94, and 0.64 in the LAO view, respectively. YOLOv5 performed the best for lesions of CTO and LS at both the image level and the patient level. In conclusion, the one-stage model without segmentation as YOLOv5 is feasible to be used in automatic coronary lesion detection, with the most suitable types of lesions as LS and CTO.

## 1. Introduction

Coronary artery disease (CAD) is one of the most common types of cardiovascular disease. It could cause stenoses and occlusions of coronary arteries, which will finally lead to severe endpoints such as myocardial ischemia and infarction. It is also the leading cause of mortality in the world, which is responsible for 16% of the total 55.4 million deaths in recent years [1]. Coronary angiography (CAG), which is recommended as the most important examination for CAD, is considered the gold standard for the diagnosis and treatment of ischemic heart disease [2,3,4]. CAG images can provide detailed anatomical information of vessels from multiple angle views, which is better than other examinations such as coronary CT angiography (CCTA) and cardiac magnetic resonance imaging (cMRI).

However, compared to CCTA and cMRI, CAG images still have some limitations: (1) Instantaneous contrast agent inhomogeneity makes the images fuzzy, with poor contrast between vessels and surrounding tissues; (2) irregular angle views cause images to change continuously; (3) complex vessel structures in two-dimensional images cause different coronary arteries to overlap and make them difficult to distinguish. Even so, given its extensive clinical application and significant diagnostic value, many studies still try to perform studies of artificial intelligence (AI)-assisted diagnosis of CAG via the deep learning (DL) method. The method of segmentation before detection has been mostly employed in previous studies. As described in the limitations of CAG images, difficulties in defining and detecting lesions caused by overlapped coronary arteries were the major challenges in the one-stage detection of multi-type coronary lesions. However, right coronary arteries rarely encounter these challenges due to less overlap.

Currently, segmenting the coronary arteries followed by diameter measurements or stenosis evaluations is the most studied method [5,6,7]. Zhao et al. [8] classified the lesions by performing image segmentation of the vessel centerline, calculating vessel diameters, and measuring the degree of stenoses. Liu et al. [9] performed vessel boundary-aware segmentation, branch node localization, coronary artery tree construction, and vessel diameter fitting, and ultimately accomplished stenosis detection. Algarni et al. [10] employed image noise removal, contrast enhancement, and Otsu thresholding as pre-processing techniques and used attention-based nested U-Net and VGG-16 for vessel segmentation and lesion detection. Their method only generated a binary classification of normal and abnormal images. However, both vessel segmentation and the extraction of coronary artery centerlines require significant work regarding manual annotation. Meanwhile, providing pixel-level specific lesion annotations for each frame reduces the robustness of lesion assessment and limits its clinical use and applications with large datasets.

Furthermore, some studies have stepped further by incorporating the automatic selection of contrast-enhanced images to extract the key frames of diagnosis for AI analysis. Cong et al. [11] employed convolutional neural networks (CNNs) and long short-term memory (LSTM) networks for automatic detection and key frame sampling. Then, they used the modified pre-trained Inception-V3 network [12] and employed the anchor-based feature pyramid network (FPN) for stenosis localization. Similarly, Moon et al. [13] used weakly supervised DL to extract key frames and performed the classification of regions of 50% stenosis. Then, they used the convolutional block attention module (CBAM) [14] to achieve the precise localization of vessel stenosis.

Some other studies have also employed multiple types of network models to improve detection performance. Ling et al. [15] used ResNet, Mask R-CNN, and RetinaNet to construct a system that includes functionalities of classification, segmentation, and detection. Du et al. [16] designed a multi-scale CNN to extract texture features of different scales from CAG images. They used the Faster R-CNN [17] framework for the detection and localization of stenoses. Danilov et al. [18] also trained and tested eight different detectors based on various network architectures and confirmed the feasibility of DL methods for the real-time detection of coronary stenoses by the intercomparisons among them.

On the other hand, studies also used artificially synthesized data because of the significant manual pre-processing steps of CAG images. Antczak et al. [19] trained a patch-based classification model with an artificial dataset and then tuned up the network using real-world patches to improve its accuracy. Ovalle-Magallanes et al. [20] proposed a pre-trained CNN model based on transfer learning for segmentation, along with fine-tuning by artificial and real-world data, to introduce a novel method for automated stenosis detection. The relevant studies are summarized in Table 1.

However, these studies still have some limitations: (1) Data in these studies are collected from patients with CAD who might undergo medical therapy or percutaneous coronary intervention (PCI) only. Lesions of them may be mild and simple, which could not represent the real world. (2) These studies lack detailed analysis of lesions as stenoses in detailed types. Du et al. [21] segmented the coronary arteries into more than 20 segments and explored various manifestations, such as stenosis, occlusion, calcification, thrombosis, and dissection. However, they did not analyze stenoses more comprehensively, of which lesions are the most common and important in clinical practice. (3) These studies all performed detection based on segmentation. Compared to direct detection, their approaches still involved more learning steps and more complex structures. Too many methods were employed to enhance model efficiency, which leaves space for further modification.

Inspired by this, we intended to develop a strategy to overcome these shortcomings in this study. We classified vascular lesions into four categories: Local stenosis, diffuse stenosis, bifurcation stenosis, and chronic total occlusion. We conducted a multi-view analysis of angiographies from candidates and adopted YOLOv5 as the key model for segmentation-free DL study of lesion detection, localization, and classification. Furthermore, we also employed the technique of gradient-weighted class activation mapping (Grad-CAM) for the visual explanations to evaluate the model performance and the feasibility of one-stage lesion detection without segmentation.

The contributions of this study are as follows:

1. This study enrolled angiography images from patients who were candidates for coronary artery bypass (CAB) surgery for the first time to evaluate the detection performance of DL techniques with complex lesions.
2. A single-stage detection model by the region-free approach was employed for the first time to detect vascular lesions directly, aiming to improve detection efficiency.
3. A more detailed classification of vascular stenoses was performed, providing a comprehensive evaluation of the network model’s performance among different types of lesions.

## 2. Materials and Methods

### 2.1. Dataset Characteristics

**Figure 1:**
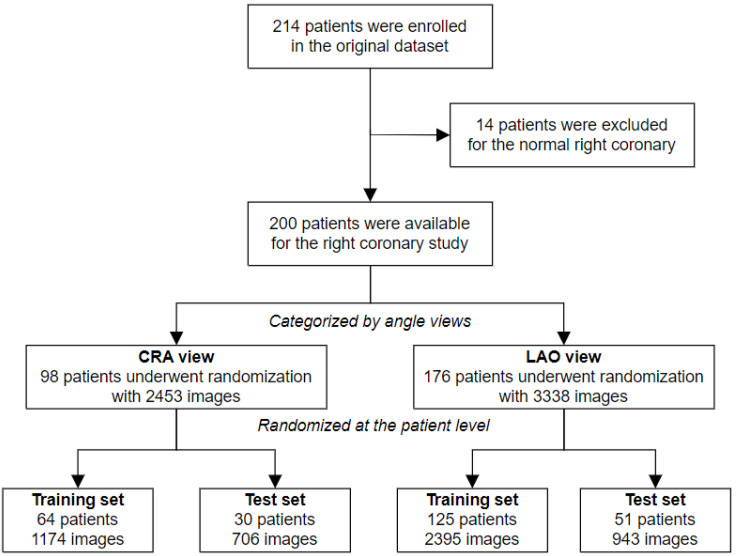
Flow chart of the study enrollment. CRA: cranial; LAO: left anterior oblique.

Two hundred and fourteen patients who were potential candidates for CAB surgery were enrolled from a single cardiac center (Fuwai Hospital, Beijing, China). This study was reviewed and approved by the ethics committee of Fuwai Hospital. There were some exclusion criteria when collecting data: (1) Combined with other cardiovascular diseases except atrial septal defect, ventricular septal defect, patent ductus arteriosus, and valvular heart disease; (2) combined with other diseases requiring surgical treatment; (3) emergency coronary artery bypass grafting or clinically unstable coronary artery disease (e.g., myocardial infarction within 30 days, preoperative implantation of the aorta counterpulsation, the need for continuous pumping of nitrates, etc.); (4) preoperative critical condition; (5) history of cardiovascular pulmonary resuscitation (CPR). The dataset was built by patients’ angiographies, which were saved as Digital Imaging and Communications in Medicine (DICOM) files and contained several angle views for left and right coronaries. Finally, images of the right coronary were analyzed in this study. Two major angle views were analyzed separately: The LAO (left anterior oblique) view is approximately 45° in the left anterior oblique view, which can display the proximal segment and middle segment well, and the CRA (cranial) view is approximately 20° in the cranial view, which can display the distal segment and posterior descending branch well. Fourteen patients had normal imaging findings with no lesion in the right coronary. Ninety-eight patients had lesions in the CRA view, and 176 patients had lesions in the LAO view. The final dataset had 2453 images in the CRA view and 3338 images in the LAO view. They were randomly divided into training sets and validation sets at the patient level by a ratio of 7:3. The enrollment profile is shown in Figure 1.

**Figure 2:**
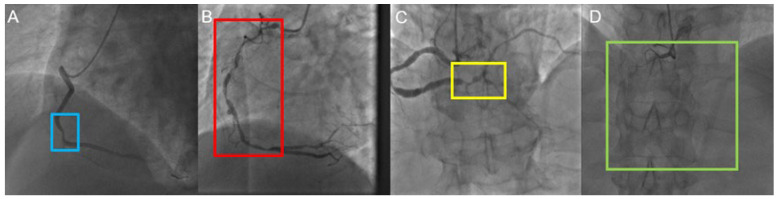
Four types of lesions on the right coronary artery. (**A**) Local stenosis (blue rectangular box); (**B**) diffuse stenosis (red rectangular box); (**C**) bifurcation stenosis (yellow rectangular box); (**D**) chronic total occlusion (green rectangular box).

Four types of lesions (Figure 2) were analyzed in this study: (1) Local stenosis (LS): A local stenosis defined as any stenosis under 20 mm in length; (2) diffuse stenosis (DS): A diffuse stenosis defined as any stenosis over 20 mm in length, which was also named long lesion [23,24]; (3) bifurcation stenosis (BS): A bifurcation stenosis defined as any stenosis adjacent to, and/or involving, the origin of a significant side branch [25]; (4) chronic total occlusion (CTO): A chronic total occlusion defined as 100% occlusion of a coronary artery for a duration of greater than or equal to 3 months based on angiographic evidence. The details of image distribution are shown in Table 2.

### 2.2. Reference Standard and Annotation Procedures

We treated manual annotations by cardiologists and radiologists as the reference standard to evaluate the diagnostic performance of the model. Firstly, a researcher converted the DICOM files into JPG image files. Then, the images of the right coronary were selected from these files and handed over to two well-trained cardiologists or radiologists with over 10 years of experience in CAG to choose ideal frames and label the lesions. The lesions were classified into four types: LS, DS, BS, and CTO. In cases of conflicting annotations, the cardiologist and the radiologist collaborated and reached a consensus to determine the final type.

### 2.3. Experimental Environment and Methodology

**Figure 3:**
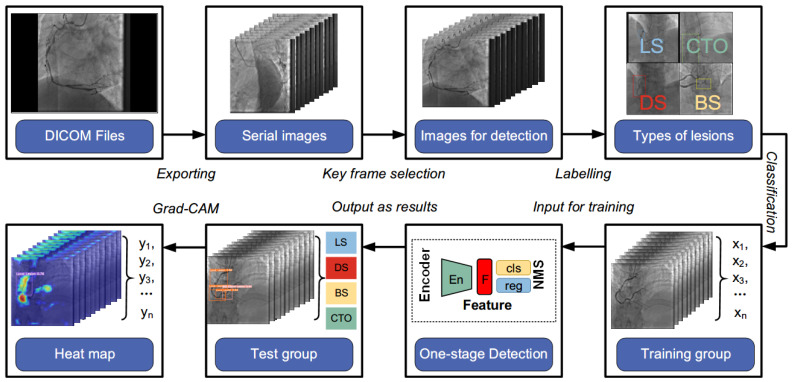
Flowchart of the proposed method. DICOM: digital imaging and communications in medicine; LS: local stenosis; DS: diffuse stenosis; BS: bifurcation stenosis; CTO: chronic total occlusion; NMS: non-max suppression; Grad-CAM: gradient-weighted class activation mapping.

Our experiments were conducted on a graphics workstation with Intel(R) Xeon Gold 6132 CPU@2.60 GHz 2.59 GHz, and NVIDIA TITAN RTX 24 G. Python 3.8 and PyTorch 1.13 were chosen as the DL framework. Figure 3 shows the flowchart of the DL procedure. DICOM Files were first exported into serial images. Ideal frames were chosen by our researcher and datasets were subsequently established. The manual annotation procedure was performed in the ways mentioned above, and the labeled images were sent to the network for training and testing. It outputs three vectors containing the predicted box class, confidence, and coordinate location in CAG images. Coronary lesions were directly detected, eliminating the requirement for time-consuming processes like segmentation and blood vessel extraction in previous studies. The types of coronary lesions were simplified to four with discriminative characteristics. To the best of our knowledge, the proposed method is the first to employ the single-stage YOLOv5 model with the region-free method to directly detect coronary lesions in CAG images. Moreover, Grad-CAM was incorporated to visualize the distinguishing area of specific lesion types for network interpretation.

We performed experiments both at the image level and the patient level. Because of the tiny changes in images in the same angle view of one single patient, it might be treated as one lesion for those found in the same position in the serial images. We defined that the prediction was correct at the patient level if one correct prediction of the lesion was found in one of the images in the serial.

### 2.4. Architecture of Models

#### 2.4.1. The YOLOv5x Model

**Figure 4:**
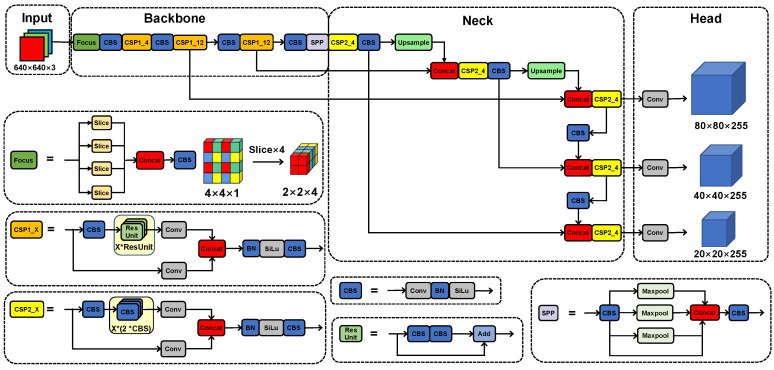
Overview of the YOLOv5x model architecture. The whole architecture contains 4 general modules, namely, an input terminal, a backbone, a neck, and a prediction network, along with 6 basic components: Focus, CSP1_X, CSP2_X, CBS, Res Unit, and SPP.

Figure 4 shows the structure of the YOLOv5x [26]. The input was uniform-size CAG image data, which were sent to the one-stage segmentation-free CNN. The network automatically learned the most class-related discriminant region highlighted to detect lesions directly, skipping the time-consuming classification and location in two steps. Finally, the network directly returned the size, position, and category of the target lesion, achieving end-to-end predictions.

The YOLOv5x consisted of a backbone feature extraction network, a neck network, and a head target prediction network. The Mosaic data enhancement method was used to augment the data, which makes the network more robust. The backbone network was mainly composed of a focus structure, a cross-stage-partial (CSP) module, and a spatial pyramid pooling (SPP) module. The focus structure sliced the input CAG images and stitched the sliced result, which reduces the loss of lesion information and effectively improves the quality of feature extraction of contrast maps. Two CSP structures were employed to speed up the inference, decrease computation, and improve lesion detection. The feature pyramid network (FPN) [27] and path aggregation network (PAN) [28] were used in the neck to realize multi-scale lesion feature fusion. Three branches of target detection heads were used in the procedure, which could detect lesions on small, medium, and large targets, respectively. The dense anchor frame could significantly increase the network’s ability to identify targets, which is obvious for small target detection. The network directly outputs results with predictions of lesion types and confidence to realize the automatic integrated prediction of the lesion type and position.

In this study, the batch size was 16 for the training set and 32 for the test set. A total of 100 epochs of training were conducted. LambdaLR was used as the learning rate updating strategy, and the stochastic gradient descent (SGD) optimizer and an initial learning rate of 10^−4^ were used. Box loss, obj (object) loss, and cls (class) loss were used:

(1) $$\begin{array}{ll} Loss=CIoULoss & +\sum_{i=0}^{S\times S}\sum_{j=0}^{B}I_{ij}^{obj}\left[C_{i}\log\left(C_{i}\right)+\left(1-C_{i}\right)\log\left(1-C_{i}\right)\right]\\ & -\sum_{i=0}^{S\times S}\sum_{j=0}^{B}I_{ij}^{noobj}\left[C_{i}\log\left(C_{i}\right)+\left(1-C_{i}\right)\log\left(1-C_{i}\right)\right]\\ & +\sum_{i=0}^{S\times S}\sum_{j=0}^{B}I_{ij}^{obj}\sum_{c\in classes}\left[p_{i}\left(c\right)\log\left(p_{i}\left(c\right)\right)+\left(1-p_{i}\left(c\right)\right)\log\left(1-p_{i}\left(c\right)\right)\right] \end{array}$$

where *S* represents the size of the final layer of feature maps and *B* is the number of detection boxes. $I_{ij}^{obj}$ stands for items in the grid $\left(i,j\right)$ and $I_{ij}^{noobj}$ for objects not present in the grid $\left(i,j\right)$.

YOLOv5 used *CIoUloss* [29] as the loss function of bounding box coordinate regression, which addresses the issue of slow convergence speed and imprecision regression in *IoU* and *GIoU* [30]. Additionally, while conducting non-maximum suppression, weighted non-maximum suppression (NMS) was employed, which effectively detects some overlapping vessels in coronary angiography images without consuming more processing resources.

#### 2.4.2. The Grad-CAM Technique

We used the Grad-CAM [31] for visual explanations after lesion detection to identify the discriminative regions in each trained model that have varied contribution weights for its classification decision. Grad-CAM can be considered mathematically as a modification of CAM and can be utilized to extend to any CNN-based network.

To understand the significance of each neuron to a specific lesion category *c* (e.g., the local stenosis), Grad-CAM used the gradient information flowing into the ultimate convolutional layer of the CNN. The neuron importance weights $\alpha_{k}^{c}$ were obtained by an averaged pooling of gradients via backpropagation from category *c*:

(2) $$\alpha_{k}^{c}=\frac{1}{Z}\sum_{i}\sum_{j}\frac{\partial y^{c}}{\partial A_{ij}^{k}}$$

where *Z* is a normalization operation. The output of Grad-CAM is generated when all feature maps of the same size are weighted and added in accordance with their respective weights. Then, a rectified linear unit (*ReLU*) was applied to the linear combination to reject feature maps with negative activation values ($A^{k}$):

(3) $$L_{Grad-CAM}^{c}=ReLU\left(\sum_{k}\alpha_{k}^{c}\cdot A^{k}\right)$$

### 2.5. Performance Evaluation

The detection performance was evaluated by the confusion matrix, precision-recall (P-R) curve, precision, recall, *F*_1_ *score*, and mean average precision (*mAP*) at the image level and the precision, recall, *F*_1_ *score*, and *mFP* at the patient level. They were defined as

(4) $$Precision=\frac{TP}{TP+FP}$$

(5) $$Recall=\frac{TP}{TP+FN}$$

(6) $$F_{1}\;score=2\times \frac{Precision\times Recall}{Precision+Recall}$$

(7) $$IoU=\frac{\left|A\cap B\right|}{\left|A\cup B\right|}$$

(8) $$mFP=\frac{FP}{n}$$

where *A* is the predicted label from YOLOv5x and *B* is the reference label. A true positive (*TP*) represents the correct classification of lesions with the intersection over union (*IoU*) ≥ threshold. A false positive (*FP*) represents the incorrect classification of lesions OR with the intersection over union (*IoU*) < threshold. The mean false positive (*mFP*) represents the mean number of *FPs* for each patient. A false negative (*FN*) is an undetected reference label. We also employed mAP@0.1 (*IoU* = 0.1) and mAP@0.5 (*IoU* = 0.5) in the study.

### 2.6. Statistics

Descriptive factors were summarized as the mean and standard deviation. Pearson’s Chi-square tests and Student’s *t*-tests were conducted for categorical and continuous factors, respectively. A two-sided *p*-value < 0.05 was considered statistically significant. Statistical Product Service Solutions (SPSS) 25.0 was used for statistical analysis.

## 3. Results

### 3.1. The Image Level

Details of the results are presented in Table 3. In the general statistics, the precision, recall, mAP@0.1, and mAP@0.5 predicted by the model were 0.64, 0.68, 0.66, and 0.49 in the CRA view, respectively. Meanwhile, the precision, recall, mAP@0.1, and mAP@0.5 predicted by the model were 0.68, 0.73, 0.70, and 0.56 in general in the LAO view, respectively. The results of CTO showed the best performance with *F*_1_ *scores* of 0.65 and 0.86 in the four types of lesions in both angle views, compared to the results of LS of 0.67 and 0.50 for the opposite.

**Figure 5:**
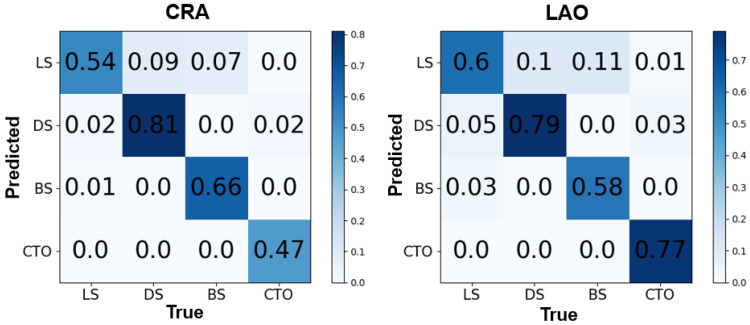
Confusion matrices of the CRA view and the LAO view. The horizontal axis represents the ground truth, and the vertical axis represents the prediction. CRA: cranial; LAO: left anterior oblique; LS: local stenosis; DS: diffuse stenosis; BS: bifurcation stenosis; CTO: chronic total occlusion.

The confusion matrices for YOLOv5x (Predicted) and manual annotations (True) of four types of lesions are shown in Figure 5 (*IoU* = 0.1). All the detected regions were taken into account when calculating the confusion matrix’s values, similar to other studies on YOLO [32,33,34]. Two angle views of the right coronary showed the same performance. In the CRA view, the probability of correct localization and classification for DS was 0.81, which was the best, and 0.54, 0.66, and 0.47 for LS, BS, and CTO, respectively. However, it was noted that 51% of the real CTO was predicted as background, while the background was also treated as LS, which represented 66% of the predicted LS. In the LAO view, the probability of correctly locating and classifying DS was 0.79, which was also the best, followed by 0.60, 0.58, and 0.77 for LS, BS, and CTO, respectively. However, like the performance in the CRA view, it could be found that 51% of the background was treated as LS in the LAO results.

**Figure 6:**
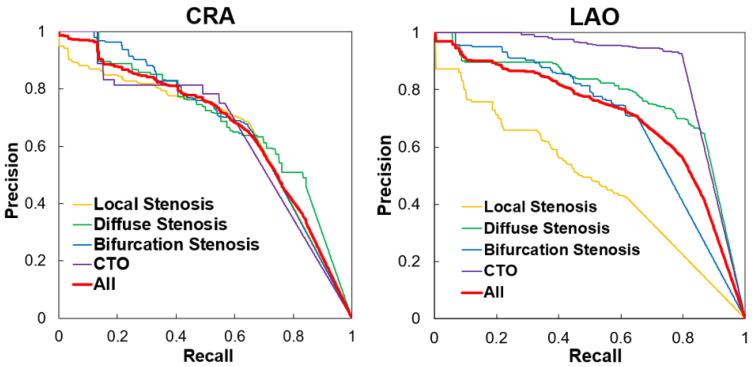
Precision-recall curves of the CRA view and the LAO view. CRA: cranial; LAO: left anterior oblique; CTO: chronic total occlusion.

The P-R curves of the two angle views shown in Figure 6 were performed for the situation of *IoU* = 0.1. The area under the curve (AUC) in general was 0.663 (mAP@0.1) in the CRA view and 0.704 (mAP@0.1) in the LAO view. It could be found in Figure 6 that in the LAO view, the result of CTO had an excellent performance, compared to the result of LS on the opposite. Meanwhile, in the CRA view, four types of lesions had the same performance.

**Figure 7:**
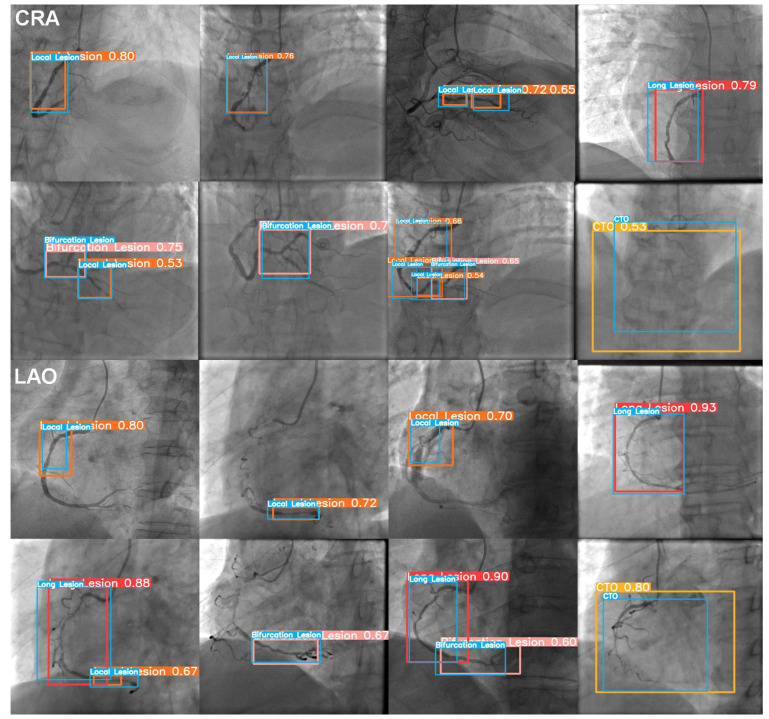
Representative coronary lesion detection results using YOLOv5 in the test set. The bounding boxes contain images of coronary lesions. CRA: cranial; LAO: left anterior oblique; Blue box: the manual annotation; Orange box: predicted local stenosis; Red box: predicted diffuse stenosis (long lesion); Pink box: predicted bifurcation stenosis; Yellow box: predicted CTO; Value: confidence.

Figure 7 shows the effect of YOLOv5x-detected lesions in CRA and LAO views. From the test results, it could be found that the model’s detection was close to the manual annotations of physicians. With the value of confidence displayed in the following, the model showed good consistency with the reference standard.

### 3.2. The Patient Level

At the patient level, the model yielded the results of the precision, recall, and *F*_1_ *score* as 0.52, 0.91, and 0.65 in the CRA view and 0.50, 0.94, and 0.64 in the LAO view, respectively. The results of CTO showed the best performance with an *F*_1_ *score* of 0.77 and 0.88 in four types of lesions in both angle views, compared to the results of 0.54 for BS and 0.44 for LS on the opposite. We also calculated the mFP in two angle views. The performance of LS made the most mistakes across the four types of lesions. The model performed the best in the CTO with 0.07 and 0.10 of mFP in both views. Moreover, the mFP was 2.47 in the CRA view and 1.86 in the LAO view. Table 4 shows the details of the results (*IoU* = 0.1).

**Figure 8:**
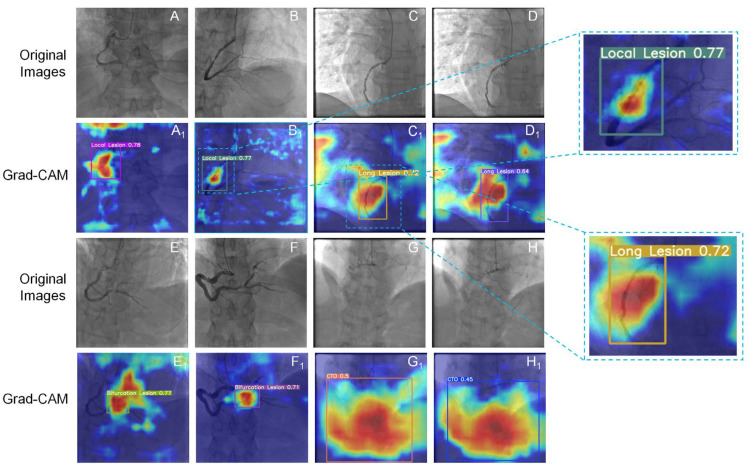
Heatmaps of Grad-CAM generated in the CRA view. The bounding boxes contain images of coronary lesions. (**A**–**H**) Original images with local stenosis (local lesion), diffuse stenosis (long lesion), bifurcation stenosis, and CTO; (**A_1_**–**H_1_**) heatmap of Grad-CAM with lesions; Value: confidence.

**Figure 9:**
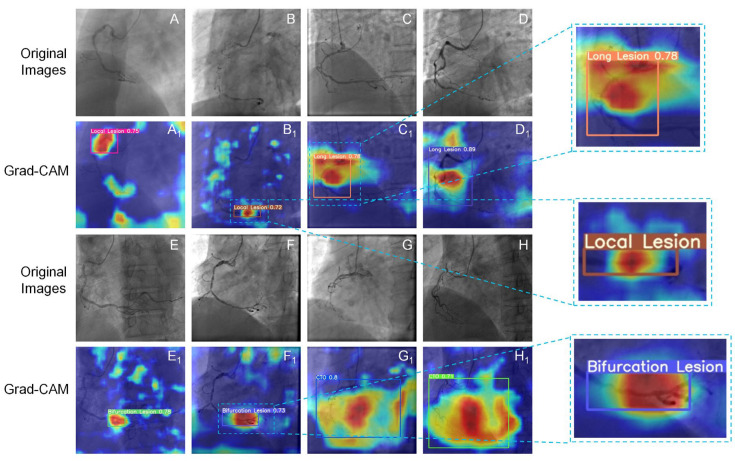
Heatmaps of Grad-CAM generated in the LAO view. The bounding boxes contain images of coronary lesions. (**A**–**H**): Original images with local stenosis (local lesion), diffuse stenosis (long lesion), bifurcation stenosis, and CTO; (**A_1_**–**H_1_**) heatmap of Grad-CAM with lesions; Value: confidence.

The Grad-CAM technique always provided valuable information on the model learning procedure. We generated the heat map of Grad-CAM to consequently testify the regions of interest for YOLOv5x in both angle views. As shown in Figure 8 and Figure 9, the activated regions (the highlighted area) corresponded to the regions that the model labeled. The model was confirmed to have a robust performance even with mild lesions. It was found that the model could learn the characteristics of lesions well and locate and classify the lesions precisely.

## 4. Discussion

This study used a single-stage model via the region-free method for the first time to detect coronary lesions directly in CAG images. We also classified common vascular abnormalities into four types: LS, DS, BS, and CTO. Our results showed that direct detection models like YOLOv5x can effectively identify vessel lesions. Meanwhile, because of the segmentation-free feature, YOLOv5x offered a more concise processing procedure, and hence it could maintain a good balance between model performance and detection efficiency in general.

In previous studies, the YOLO series of models have mostly been applied in tumor detection and retinal fundus disease evaluation. However, the fundus vessel lesion evaluation shows similarity compared to the coronary stenoses during the DL processing procedure [35,36,37]. Santos et al. [36] also used YOLOv5 as the detection model. In their public datasets of diabetic retinopathy images, YOLOv5 generated mAP@0.5 of 0.154 and an *F*_1_ *score* of 0.252. In our study, the detection of lesions achieved a precision of 0.675, a recall rate of 0.734, an mAP@0.1 of 0.558, and an *F*_1_ *score* of 0.703 in the LAO view at the image level. Meanwhile, at the patient level, the detection of lesions reached a precision of 0.792, a recall rate of 100%, an *F*_1_ *score* of 0.884, and a maximum mFP of 0.466.

Generally, it can be found that the YOLO series of models demonstrates promising performance in the automatic detection of coronary artery lesions. The high precision and recall rates at both the image and patient levels indicate the model’s reliability in identifying vascular abnormalities in CAG images. The impressive *F*_1_ *scores* further validate the model’s ability to balance precision and recall effectively. The low mFP also suggests that the model minimizes false-positive detections, which is crucial for accurate diagnosis and reducing unnecessary interventions. Overall, these findings highlight the potential of using YOLO-based direct detection models for the efficient and reliable detection of coronary artery abnormalities in medical imaging applications.

In the subgroup analysis of the four lesions, the CTO group and the DS group showed good results. They achieved a precision of 0.927, a recall rate of 0.796, mAP@0.1 of 0.870, and an *F*_1_ *score* of 0.857 for the CTO group in the LAO view at the image level and a precision of 0.648, a recall rate of 0.868, mAP@0.1 of 0.773, and an *F*_1_ *score* of 0.742 for the DS group. Du et al. [16] tested the performances of four models (CALD-Net, ZF-Net+Faster R-CNN, VGG+Faster R-CNN, and ResNet50+Faster R-CNN), finding recall rates of 0.88, 0.41, 0.50, and 0.62. Pang et al. [22] tested the performances of five models (Faster R-CNN, Guided Anchoring, Libra R-CNN, Cascade R-CNN, and Stenosis-DetNet), finding *F*_1_ *scores* of 0.80, 0.79, 0.81, 0.78, and 0.88. Even in the analysis with a large dataset comprising 20,612 CAG images of 10,073 patients, it had a precision of 0.769 for the stenosis and 0.757 for the CTO lesion [21]. Our study showed that the direct detection of lesions like CTO and diffuse stenoses had the same performance compared to these studies. Consequently, it might be concluded that single-stage detection models like YOLOv5 could generate a stable result, which is similar to, or even better than, detection models combining segmentation in suitable situations.

However, in our study, the performance in the LS group showed an unsatisfactory result. In the LAO view of the image level, the LS group had a precision of 0.426, a recall rate of 0.617, a mAP@0.1 of 0.479, and an *F*_1_ *score* of 0.504. At the patient level, the LS group also had the highest mFP compared to other groups with results of 1.467 in the CRA view and 1.118 in the LAO view, which meant more than one false labeling of LS for each patient. Correspondingly, the mFP in the CTO group was just 0.067 in the CRA view and 0.098 in the LAO view. Moon et al. [13] used the internal dataset and external dataset in their study. They showed a similar performance, with a mean accuracy of diffuse lesions better than focal lesions in each dataset. These results might be related to factors such as low-range stenosis, which is inconspicuous, susceptibility to background noises, and small lesion characteristics resulting in confusion with the visual features of normal arteries. Therefore, it is necessary to perform segmentation before the detection of local stenoses in the DL procedure.

Grad-CAM demonstrated the network-learned lesion characteristics, located the identification details of lesions, and visualized the distinguishing area of specific lesion types in the image based on DL. The low-heat region and high-heat region in the heatmap are determined based on the contribution of the regions in the image to the identification of lesions, with the high-heat region playing a decisive part in the network’s inferential decision-making. The network has successfully learned the characteristics of the lesion, allowing the lesion area to receive adequate attention in Grad-CAM, as indicated by the position of the intact area with high heat (darker part) and the detection box being consistent. Figure 8B_1_ and Figure 9B_1_ show that the model effectively learned the tiny characteristics of local stenoses and classified them correctly. Moreover, high-heat areas were only visible in the stenosis area but not in normal blood vessels. As can be observed in the wide array of high-heat areas in Figure 8G_1_,H_1_ and Figure 9G_1_,H_1_, CTO exhibited a greater range of characteristics than local stenosis, which was also identified by the model. However, Grad-CAM struggles to show only the complicated regions that require attention. Some noise might be produced, which manifests as comparatively low-heat areas like the edge regions in C1 of Figure 8.

This study has several limitations. (1) We only performed the DL analysis in the right coronary. Lesions in the right coronary are always simpler than in the left. The YOLO series of models might face much bigger challenges, and their robustness should be tested in more complex circumstances. (2) The CAG images of candidate patients were collected in primary hospitals in our country, which might make it difficult to control the quality of angiography. It could be an important confounding factor that would impact the final performance of network models. (3) Our dataset should be enriched in future studies. The YOLOv5 model performed better for the local stenosis in the CRA view than for the CRA view, accompanied by a dataset of 1055 lesions compared to 433 lesions. It could be supposed that the performance of YOLOv5 could be better in a huge dataset of CAG images.

## 5. Conclusions

Our study used the one-stage strategy to detect coronary lesions in a segmentation-free manner and demonstrated that the YOLOv5 model could be feasible in CAG analysis using the DL method, with good robustness. We also found in the subgroup study that lesions of CTO and DS were most suitable for direct detection without segmentation, which could shorten processing time and improve working efficiency.

## Figures and Tables

**Table 1:** Related studies are summarized in four aspects: Methods, data, classes, and results.

Ref.	Methods	Data	Classes	Results
Zhao et al. (2021) [8]	FP-U-Net++, arterial centerline extraction, diameter calculation, arterial stenosis detection	99 patients, 314 images	1–24%, 25–49%, 50–69%, 70–100%	Precision = 0.6998, recall = 0.6840,
Liu et al. (2023) [9]	AI-QCA	3275 patients, 13,222 images	0–100%	Precision = 0.897, recall = 0.879
Algarni et al. (2022) [10]	ASCARIS model	130 images	normal and abnormal	Accuracy = 97%, recall = 95%, specificity = 93%
Cong et al. (2023) [11]	Inception-v3 and LSTM, redundancy training, and Inception-V3, FPN	230 patients, 14,434 images	<25%, 25–99%, CTO	Accuracy = 0.85, recall = 0.96, AUC = 0.86
Moon et al. (2020) [13]	GoogleNet Inception-v3, CBAM, Grad-CAM	452 clips	Stenosis ≥ 50%	AUC = 0.971, accuracy = 0.934
Ovalle-Magallanes et al. (2020) [20]	pre-trained CNN via Transfer Learning, CAM	10,000 artificial images, 250 real images	Stenosis	Accuracy = 0.95, precision = 0.93, sensitivity = 0.98, specificity = 0.92, $F_{1}\;score$ = 0.95
Antczak et al. (2021) [19]	A patch-based CNN for stenosis detection	10,000 artificial images, 250 real images	Stenosis	Accuracy = 90%
Du et al. (2021) [21]	A DNN for the recognition of lesion morphology	10,073 patients, 20,612 images	Stenotic lesion, total occlusion, calcification, thrombus, and dissection	$F_{1}\;score$ = 0.829, 0.810, 0.802, 0.823, 0.854
Ling et al. (2023) [15]	DLCAG diagnose system	949 patients, 2980 images	Stenosis	mAP = 86.3%
Danilov et al. (2021) [18]	Comparison of state-of-the-art CNN (N = 8)	100 patients, 8325 images	Stenosis ≥ 70%	mAP = 0.94, *F*_1_ *score* = 0.96, prediction speed = 10 fps
Pang et al. (2021) [22]	Stenosis-DetNet with SFF and SCA	166 sequence, 1494 images	Stenosis	Accuracy = 94.87%, sensitivity 82.22%

**Table 2:** Distributions of images and lesions in the CRA and LAO angle views.

	The CRA View	The LAO View	*p* Value
Age, years	63 ± 8	64 ± 9	0.54
Gender			
Male (%)	68 (69%)	118 (67%)	0.72
Images	2453	3338	0.66
Training Set (%)	1747	2395	
Test Set (%)	706	943	
Lesions			
Training Set	3259	1529	<0.01
LS	2003	1005	
DS	376	96	
BS	500	375	
CTO	380	53	
Test Set	3874	1262	<0.01
LS	2187	433	
DS	405	273	
BS	411	174	
CTO	871	382	

CRA: cranial; LAO: left anterior oblique; LS: local stenosis; DS: diffuse stenosis; BS: bifurcation stenosis; CTO: chronic total occlusion.

**Table 3:** Results of four lesions with two angle views at the image level.

	Lesions	Number	Precision	Recall	mAP@0.1	mAP@0.5	*F*_1_ *Score*
CRA	LS	1055	0.685	0.647	0.643	0.405	0.665
	DS	96	0.458	0.844	0.687	0.677	0.594
	BS	374	0.656	0.658	0.675	0.625	0.657
	CTO	53	0.75	0.566	0.647	0.263	0.645
	All	1578	0.637	0.679	0.663	0.493	0.657
LAO	LS	433	0.426	0.617	0.479	0.273	0.504
	DS	273	0.648	0.868	0.773	0.688	0.742
	BS	174	0.699	0.655	0.694	0.521	0.676
	CTO	382	0.927	0.796	0.87	0.749	0.857
	All	1262	0.675	0.734	0.704	0.558	0.703

mAP@0.1: mean average precision (*IoU* = 0.1); mAP@0.5: mean average precision (*IoU* = 0.5); CRA: cranial; LAO: left anterior oblique; LS: local stenosis; DS: diffuse stenosis; BS: bifurcation stenosis; CTO: chronic total occlusion.

**Table 4:** Results of four lesions with two angle views at the patient level.

	Lesions	*TP* + *FN*	*TP*	*FN*	*FP*	P	R	*F*_1_ *Score*	*mFP*
CRA	LS	59	55	4	44	0.556	0.932	0.696	1.467
	DS	6	6	0	8	0.429	1.000	0.600	0.267
	BS	15	13	2	20	0.394	0.867	0.542	0.667
	CTO	6	5	1	2	0.714	0.833	0.769	0.067
	All	86	79	7	74	0.523	0.908	0.652	2.467
LAO	LS	28	24	4	57	0.296	0.857	0.440	1.118
	DS	18	18	0	17	0.514	1.000	0.679	0.333
	BS	11	10	1	16	0.385	0.909	0.541	0.314
	CTO	19	19	0	5	0.792	1.000	0.884	0.098
	All	76	71	5	95	0.497	0.942	0.636	1.863

*TP*: true positive; *FN*: false negative; *FP*: false positive; P: precision; R: recall; *mFP*: mean predicted positive; CRA: cranial; LAO: left anterior oblique; LS: local stenosis; DS: diffuse stenosis; BS: bifurcation stenosis; CTO: chronic total occlusion.

## Data Availability

The raw data supporting the conclusions of this article may be provided upon reasonable requests for scientific research purposes.

## Abbreviations

The following abbreviations are used in the manuscript:

AI	Artificial Intelligence
BS	Bifurcation Stenosis
CAB	Coronary Artery Bypass
CAD	Coronary Artery Disease
CAG	Coronary AngioGraphy
CNN	Convolutional Neural Network
CPR	Cardiovascular Pulmonary Resuscitation
CRA	CRAnial
CTO	Chronic Total Occlusion
DICOM	Digital Imaging and COmmunications in Medicine
DL	Deep Learning
DS	Diffuse Stenosis
FN	False Negative
FP	False Positive
Grad-CAM	Gradient-weighted Class Activation Mapping
IoU	Intersection over Union
LAO	Left Anterior Oblique
LS	Local Stenosis
mAP	mean Average Precision
mFP	mean False Positive
PCI	Percutaneous Coronary Intervention
PR	Precision-Recall
TN	True Negative
TP	True Positive
